# Detecting type 2 diabetes and prediabetes among asymptomatic adults in the United States: modeling American Diabetes Association versus US Preventive Services Task Force diabetes screening guidelines

**DOI:** 10.1186/1478-7954-12-12

**Published:** 2014-05-07

**Authors:** Timothy M Dall, K M Venkat Narayan, Karin B Gillespie, Paul D Gallo, Tericke D Blanchard, Mihaela Solcan, Michael O’Grady, William W Quick

**Affiliations:** 1 IHS Life Sciences, Washington, DC, USA; 2 Rollins School of Public Health, Emory University, Atlanta, GA, USA; 3 Novo Nordisk Inc., Plainsboro, NJ, USA; 4 University of Chicago, Chicago, IL, USA; 5 D-is-for-Diabetes.com, North Charleston, SC, USA

**Keywords:** Type 2 diabetes, Prediabetes, Screening, American Diabetes Association, USPSTF

## Abstract

**Background:**

Screening to detect prediabetes and diabetes enables early prevention and intervention. This study describes the number and characteristics of asymptomatic, undiagnosed adults in the United States who could be detected with prediabetes and type 2 diabetes using the American Diabetes Association (ADA) guidelines compared to the United States Preventive Services Task Force (USPSTF) guidelines.

**Methods:**

We developed predictive models for undiagnosed diabetes and prediabetes using polytomous logistic regression from data on risk factors in the 2003–2010 National Health and Nutrition Examination Survey (n = 19,056). We applied these predictive models to the 2010 Medical Expenditure Panel Survey, which contains health care use data, to generate probabilities of undiagnosed diabetes and undetected prediabetes for each adult. We summed individual probabilities to estimate the number of adults who would be detected with prediabetes and/or type 2 diabetes if screened under ADA or USPSTF guidelines. We analyzed health care use patterns of people at high risk for diabetes.

**Results:**

In 2010, 59.1 million adults met the USPSTF screening criteria including 24.4 million people with undetected prediabetes and 3.7 million people with undiagnosed diabetes. In comparison, among the 86.3 million people who met the ADA screening criteria, there were 33.9 million with undetected prediabetes and 4.6 million with undiagnosed type 2 diabetes. The ADA guidelines detected 38.9% more cases of prediabetes and 24.3% more cases of type 2 diabetes compared to the USPSTF guidelines. Subgroup analysis showed that ADA guidelines would detect 78% more cases of diabetes among the age 54 and younger population, in 40% more blacks, and in more than twice as many Hispanics than USPSTF guidelines. Only 58% of adults meeting ADA guidelines and 70% meeting USPSTF guidelines had ≥ 1 primary care office visit in 2010.

**Conclusions:**

Compared to USPSTF guidelines, ADA guidelines would screen more people and detect more cases of both prediabetes and type 2 diabetes, though a substantial percentage of patients with undetected cases had no contact with a primary care provider in 2010. Addressing the problem of large numbers of undetected prediabetes and type 2 diabetes cases will require new strategies for screening.

## Background

Type 2 diabetes is a large, costly, and growing epidemic in the US [1]. Evidence-based interventions are available to prevent or delay the onset of diabetes in people with prediabetes [2,3] and to reduce rates of complications among those with type 2 diabetes [4]. More than one-fourth of the estimated 26 million Americans with diabetes remain undiagnosed, and more than 90% of the estimated 79 million adults with prediabetes remain undetected [5]. As with many diseases, screening and early detection of diabetes and prediabetes is the first step to initiating prevention and treatment interventions, and have received considerable interest [6-8]. Screening is recommended within a health care setting, usually by a primary care provider, so that appropriate follow-up testing and care can be delivered [4].

US and international organizations have recommended various guidelines for screening for type 2 diabetes in asymptomatic adults, but these guidelines differ in the number and types of risk factors they target [4,8-12]. The United States Preventive Services Task Force (USPSTF), an independent panel of experts that conducts scientific reviews of preventive health care services, recommends screening only adults with sustained hypertension (either treated or untreated) greater than 135/80 mm Hg [8]. In comparison, the American Diabetes Association (ADA) recommends broader screening criteria by targeting everyone age 45 and older as well as overweight adults of any age who have additional risk factors, including family history of diabetes, being a member of a high-risk racial/ethnic population, physical inactivity, high cholesterol, signs of insulin resistance (such as acanthosis nigricans), polycystic ovarian syndrome, history of gestational diabetes, or previous diagnosis of prediabetes, as well as having hypertension [4]. For adults age 45 and older with normal test results, the ADA recommends repeat testing at least every three years. Other organizations have also endorsed guidelines that encompass multiple risk factors to screen for asymptomatic adults—including the American Heart Association, the American College of Physicians, The Endocrine Society, and the Veterans Health Administration [13-16]. While USPSTF guidelines are designed to detect diabetes, ADA guidelines are designed to detect both diabetes and prediabetes.

The number of people screened and detected with diabetes or prediabetes in a given population will differ depending on which guidelines are followed. For example, analysis of an ambulatory population seen at one large physician practice found following the ADA guidelines would detect 50% more cases of undiagnosed diabetes than would be detected if following the USPSTF guidelines [17]. A study modeling simulated screening strategies found that screening based on USPSTF guidelines identified fewer people with diabetes compared to screening initiated at age 45, as recommended by the ADA [18].

Our study investigates two sets of questions with respect to USPSTF and ADA screening guidelines: (1) How many people in the US in 2010 could have been screened and identified with diabetes and prediabetes under each set of guidelines, and what are the characteristics of populations detected with prediabetes and diabetes? (2) What are the health care use patterns of adults at high risk for prediabetes or diabetes, and how does this affect the ability to implement USPSTF and ADA guidelines?

## Methods

### Data sources

We used the 2003–2010 waves of the National Health and Nutrition Examination Survey (NHANES), a major nationally representative survey of the US non-institutionalized population, to develop predictive models for undiagnosed diabetes and undetected diabetes [19]. NHANES includes detailed health information and characteristics, including whether a respondent has ever been told by a health care professional that he or she has diabetes or prediabetes. A random sample of approximately one-third of NHANES adults receives laboratory tests to provide more detailed descriptions of their health status. Comparison of self-reported glycemic status with laboratory tests provides an opportunity to develop a clinically based model of undiagnosed glycemic disease. These laboratory tests include hemoglobin A1c (HbA1c), fasting plasma glucose (FPG), and/or oral glucose tolerance test (OGTT). Many individuals receive more than one test type.

We used the 2010 Medical Expenditure Panel Survey (MEPS) to analyze health care use patterns and estimate the number of people whose diabetes or prediabetes could be detected using the ADA and USPSTF screening guidelines [20]. Like NHANES, MEPS collects detailed information on patient characteristics, health-related behavior, and presence of chronic conditions. MEPS does not contain lab values but does collect detailed information on health care use patterns for each participant over a one-year period. MEPS contains a self-reported indication of previous diabetes diagnosis, but includes no data on previous prediabetes diagnosis. Both surveys contain sample weights to generalize from the sample to the US population, and weights were used in the regression analyses and to generate summary statistics.

### Selection and exclusion criteria

Exclusion criteria for our analysis included women who indicated they were pregnant at the time of their NHANES lab test (n = 425), as well as people who indicated that they had previously been told by a health care professional that they have diabetes or for whom there was an indication of treatment for diabetes (n = 2,300). This provides a sample of 19,056 individuals with lab results— including 9,855 who received only the HbA1c test; 5,809 who received all three tests (HbA1c, FPG, and OGTT); 3,362 who received both HbA1c and FPG; 18 who received FPG and OGTT; seven who received only FPG; and five who received both HbA1c and OGTT. We applied similar exclusion criteria for the MEPS analysis, restricting the population to non-pregnant adults age 18 and older without diagnosed diabetes (n = 21,774).

### Definitions of key variables

Using lab values in NHANES, diabetes was defined as OGTT ≥ 200 at two hours or FPG ≥ 126 or HbA1c ≥ 6.5; prediabetes was defined as 199 ≥ OGTT ≥ 140 at two hours, 125 ≥ FPG ≥ 100, or 6.4 ≥ HbA1c ≥ 5.7 [4]. We categorized people as having diabetes if any of their lab tests were in the diabetes range. For individuals not categorized as having diabetes, we categorized them as having prediabetes if any of their test results were in the prediabetes range. A limitation of NHANES, discussed later, is that no follow-up confirmatory test is available, and research suggests that using a single test can result in false positives or negatives [21].

For modeling purposes, we placed each NHANES adult into one of four categories: undiagnosed diabetes, diagnosed prediabetes, undetected prediabetes, or normal lab levels. Undiagnosed diabetes and undetected prediabetes were defined by a negative response to the question “have you ever been told by a health care professional that you have diabetes or prediabetes?” and a positive finding for diabetes or prediabetes on the lab test results [22].

We selected explanatory variables for the regression analysis (described later) based on established [9,10,23-27] risk factors for diabetes that are common to the NHANES and MEPS databases, as well as variables associated with greater access to or use of health care services. All variables were coded as dichotomous indicators (characteristic applies = 1, else = 0), with the exception of family income (measured continuously in thousands of 2010 dollars). Variables used were sex; six age groups (18–34, 35–44, 45–54, 55–64, 65–74, and 75+ years); race/ethnicity (non-Hispanic white, non-Hispanic black, non-Hispanic other, and Hispanic); previous diagnoses or history of asthma, arthritis, heart attack, stroke, cancer, hypertension, high cholesterol, and cardiovascular disease; current smoker; body weight defined by body mass index [28]—normal (BMI < 25), overweight (25 ≤ BMI < 30), or obese (30 ≤ BMI); has medical insurance; is insured through Medicaid; and survey year. While arthritis and asthma are not recognized risk factors for diabetes, we included these indicators because patients with these conditions have more annual visits with health care providers, which could increase the number of opportunities for screening. Including these two conditions might also result in earlier identification of diabetes or prediabetes if such patients were treated with corticosteroids, in light of the known hyperglycemic effect of these medications. Our analysis omitted ADA screening risk factors for which data are unavailable in NHANES or MEPS (family history of diabetes, polycystic ovarian syndrome, or gestational diabetes).

### Statistical analysis plan

Using NHANES data for those individuals not previously diagnosed with diabetes (n = 19,056), we estimated a polytomous logistic predictive model. This regression approach allowed us to model a dependent variable with three values: normal glucose levels, prediabetes (both diagnosed and undiagnosed), and undiagnosed diabetes, and thus provided estimated risks for both diabetes and prediabetes [29]. We applied this predictive model from NHANES to each adult in MEPS to generate individual probabilities of undiagnosed diabetes and prediabetes based on each person’s demographic, health, and socioeconomic characteristics.

Separate from the polytomous logistic regression, we used logistic regression to quantify the relationship between patient characteristics and diagnosed prediabetes (n = 676). We used the same explanatory variables as described previously, with the dependent variable indicating previous detection of prediabetes. We applied this second regression to the MEPS sample to estimate each person’s probability of previous prediabetes detection. The total probability of prediabetes minus the probability of detected prediabetes provided an estimated probability of undetected prediabetes for each person.

We then identified adults in the MEPS who would be screened under the USPSTF and ADA screening guidelines and summed their predicted probabilities for undetected prediabetes and undiagnosed diabetes to provide estimates of the potential number of people in the US who could be detected under each screening guideline. Consider, for example, an individual with a predicted probability of 0.3 for undetected prediabetes and 0.1 for undiagnosed diabetes, and with a sample weight of 1000 (meaning this person represents 1,000 people in the US population). If this sample person met the screening criteria, then he or she represents 1,000 people screened, 300 people (0.3 × 1,000) in whom prediabetes would be detected, and 100 people (0.1 × 1,000) in whom diabetes would be diagnosed.

When modeling the ADA screening guidelines (using those risk factors available in MEPS), if a person met the criteria only because he or she was over age 45 (that is, was not overweight or had no other risk factors), we modeled this person as having a one-in-three probability of being screened during the year. This assumption was to simulate the ADA recommendation that a person over age 45 with no risk factors should be re-screened every three years.

We then used MEPS to analyze health care use patterns of people who met the ADA and USPSTF screening criteria to estimate the number of detection (screening) opportunities that exist under current patterns of usage.

To validate the predictive modeling approach, we randomly divided the NHANES sample into two groups with 9,528 observations each. We estimated a predictive model for prediabetes and undiagnosed diabetes with one group, and then applied the model to the second group. For the second group, we compared the sum of predicted probabilities of prediabetes and undiagnosed diabetes with the clinical indication of prediabetes or undiagnosed diabetes. The analysis suggests that the predictive modeling approach reliably estimated total cases of prediabetes and undiagnosed diabetes in the population by age group. Regressions estimated with both subsets of NHANES produced similar coefficients, so we used the full NHANES sample to generate the results presented in this paper.

## Results

### NHANES sample and predictive model

Summary statistics for the NHANES sample are consistent with the published literature and are summarized in Table 1. Approximately 90% of prediabetes cases were undetected. Characteristics associated with higher odds of having undiagnosed diabetes or prediabetes include male, older age, minority race and Hispanic, hypertension, hypercholesterolemia, cardiovascular disease, smoking, and excess body weight (Table 2) [5]. Having medical insurance and higher annual family income are associated with lower odds of prediabetes and undiagnosed diabetes. Many of the factors associated with prediabetes and undiagnosed diabetes are the same as those associated with diagnosed diabetes. Variation across NHANES years could be due in part to changes in laboratory methodology across different NHANES years.

**Table 1:** NHANES descriptive statistics by diabetes population (% or $)

**Patient characteristics**	**Normal glucose levels (n = 11,520)**	**Undiagnosed prediabetes (n = 5,804)**	**Diagnosed prediabetes (n = 676)**	**Undiagnosed diabetes (n = 1,056)**	**Diagnosed diabetes (n = 2,296)**
Male	45	55	42	55	50
Age category					
Age 18-34	47	17	11	5	4
Age 35-44	18	15	15	12	7
Age 45-54	13	19	18	16	16
Age 55-64	9	18	21	21	26
Age 65-74	6	15	20	23	28
Age 75+	7	15	16	24	19
Race and ethnicity					
Non-Hispanic white	73	68	72	68	63
Non-Hispanic black	10	12	11	11	16
Non-Hispanic other	5	7	7	5	7
Hispanic	12	13	9	15	13
Has been diagnosed with					
Asthma	13	12	19	13	16
Arthritis	17	30	46	37	49
History of heart attack	2	5	8	8	12
History of stroke	2	4	5	5	11
History of cancer/malignancy	6	10	15	14	14
High blood pressure	19	38	56	54	70
High cholesterol	18	33	50	40	57
Cardiovascular disease	3	8	13	14	22
Current smoker	21	21	16	20	17
Body weight status	41	25	20	17	17
Normal	41	25	20	17	17
Overweight	33	36	31	31	28
Obese	26	39	50	52	55
Median annual family income (thousands of US$)	40	35	30	30	30
Has medical insurance	74	76	85	80	87
Insured through Medicaid	5	6	8	7	10
NHANES survey wave					
Years 2003-4	28	17	9	14	21
Years 2005-6	26	20	22	19	20
Years 2007-8	22	31	33	34	30
Years 2009-10	25	32	36	33	29

Source: Analysis of 2003–2010 National Health and Nutrition Examination Survey. Note: Body weight status is defined by body mass index (BMI): normal (BMI < 25), overweight (25 ≤ BMI < 30), and obese (30 ≤ BMI).

**Table 2:** Odds ratios from NHANES regressions

**Effect**	**Undiagnosed diabetes and total prediabetes**^**†**^	**Diagnosed prediabetes**^**ŧ**^	**Diagnosed diabetes**^**ŧ**^
Male	1.44*	0.80*	1.16*
Age 18–34 (comparison)			
Age 35-44	1.93*	2.01*	2.09*
Age 45-54	3.70*	2.04*	4.12*
Age 55-64	5.52*	2.50*	5.46*
Age 65-74	7.03*	2.43*	7.10*
Age 75+	8.89*	1.90*	5.79*
Non-Hispanic white (comparison)			
Non-Hispanic black	1.61*	1.07	1.92*
Non-Hispanic other	1.97*	1.63*	2.23
Hispanic	1.61*	1.08	2.22*
Has been diagnosed with			
Asthma	1.05	1.51*	1.10*
Arthritis	1.02	1.45*	1.22*
History of heart attack	1.00	1.47	1.11
History of stroke	1.06	1.07	1.82*
History of cancer/malignancy	1.03	1.19	1.07
High blood pressure	1.36*	1.81*	2.15*
High cholesterol	1.18*	1.56*	1.90*
Cardiovascular disease	1.18*	1.14	1.87*
Current smoker	1.17*	0.72*	0.95
Overweight vs. normal weight	1.60*	1.30*	1.22*
Obese vs normal weight	2.96*	1.92*	2.79*
Annual family income (thousands of US$)	0.998*	0.998	0.994*
Has medical insurance	0.81*	1.16	1.22
Insured through Medicaid	1.17	1.11	1.24*
Survey Years 2003–4 (comparison)			
Years 2005-6	1.42*	2.86*	0.99
Years 2007-8	2.43*	3.39*	1.09
Years 2009-10	2.20*	3.79*	1.04
Sample size	509/7,027/11,520 UDM/PDM/normal	676/18,380 PDM/non-diabetic	2,296/19,056 DDM/non-DDM
Model goodness-of-fit statistics			
Percent concordant	76.2	76.1	83.0
Percent discordant	23.5	22.5	16.6
Percent tied	0.4	1.4	0.4
Number of pairs matched	90,391,463	12,424,880	43,752,576
Somers’ D	0.529	0.535	0.664
c-statistic	0.764	0.768	0.832

Source: Analysis of 2003–2010 National Health and Nutrition Examination Survey. Notes: Body weight status is defined by body mass index (BMI): normal (BMI < 25), overweight (25 ≤ BMI < 30), and obese (30 ≤ BMI). ^†^ Polytomous logistic regression. ^ŧ^ Usual logistic regression. * Odds ratio statistically different from 1.0 at the 0.05 level. DDM = diagnosed diabetes, UDM = undiagnosed diabetes, PDM = prediabetes.

Applying the predictive model to MEPS adults who do not have diagnosed diabetes produced probabilities of undiagnosed diabetes ranging from 0.3% to 26.2%. Whereas the person among the MEPS sample with the lowest predicted probability of undiagnosed diabetes is a young, non-Hispanic white, high-income female with no history of chronic conditions and no known risk factors for diabetes, the person with the highest predicted probability is older, male, non-Hispanic other (non-black) minority, obese, and with a history of hypertension, high cholesterol, and cardiovascular disease.

**Figure 1:**
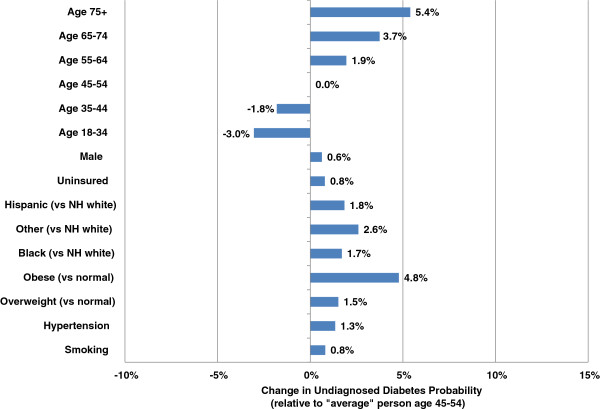
**Standardized effect on probability of undiagnosed diabetes controlling for other patient characteristics.** Note: This figure is based on patient risk for undiagnosed diabetes assuming population averages for all characteristics except the characteristic of interest (where the presence of the characteristic is compared to the absence of that characteristic).

**Figure 2:**
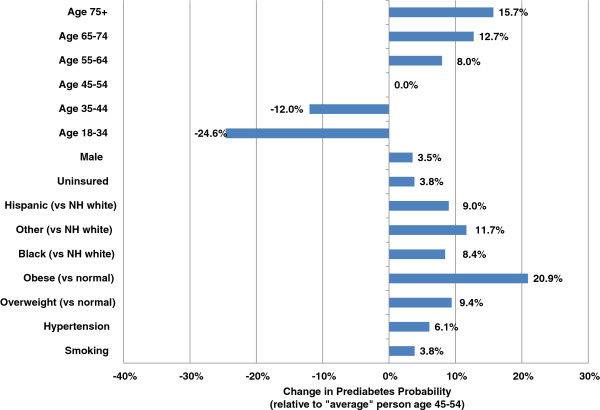
**Standardized effect on probability of prediabetes and diabetes controlling for other patient characteristics.** Note: This figure is based on patient risk for prediabetes assuming population averages for all characteristics except the characteristic of interest (where the presence of the characteristic is compared to the absence of that characteristic).

Characteristics associated with statistically higher probability of undiagnosed diabetes (Figure 1) and prediabetes (Figure 2) include older age, excess body weight, racial or ethnic minority, male, hypertension, cardiovascular disease, dyslipidemia, and smoking. These characteristics substantially overlap risk factors in the ADA guidelines. Being obese (versus normal weight) is associated with a 4.8 percentage point increase in probability of undiagnosed diabetes among a population age 45 to 54 with population mean values for the other risk factors.

### USPSTF vs. ADA guidelines: Diabetes and prediabetes cases detected

The US had approximately 7 million adults with undiagnosed diabetes and 79 million with undetected prediabetes in 2010 [5]. Our analysis suggests that 59.1 million adults in the US meet the USPSTF screening guidelines, and screening of all these individuals would detect 3.7 million people with diabetes and 24.4 million with prediabetes—or about half (53%) the cases of undiagnosed diabetes and one-third (31%) of the cases of undetected prediabetes (Tables 3 and 4). Such findings are consistent with research suggesting that approximately half of the people with undiagnosed diabetes do not meet USPSTF screening guidelines [12]. In contrast, 86.3 million adults meet ADA guidelines (assuming that one in three adults age 45 or older without other risk factors were screened in a given year), and screening these adults would detect 4.6 million people with diabetes and 33.9 million people with prediabetes.

**Table 3:** Characteristics and number of adults with diabetes detected under ADA and USPSTF guidelines (thousands of people)

**Patient characteristics**	**ADA alone**	**USPSTF alone**	**Either ADA or USPSTF**	**ADA but not USPSTF**	**USPSTF but not ADA**
Age category					
<45	770	324	791	467	22
45-54	955	643	1,015	372	60
55-64	1,283	1,039	1,397	358	115
65+	1,631	1,729	2,021	292	390
Sex					
Men	2,661	2,039	2,924	885	264
Women	1,978	1,696	2,300	604	322
Race and ethnicity					
Non-Hispanic white	2,757	2,519	3,161	642	404
Non-Hispanic black	831	589	900	311	69
Non-Hispanic other	214	237	278	42	64
Hispanic	837	391	886	494	49
Body weight status					
Overweight	2,036	1,298	2,036	739	0
Obese	2,603	1,852	2,603	750	0
Diagnosed hypertension	3,150	3,736	3,736	0	586
Household income quartile					
First	1,073	823	1,217	352	136
Second	1,087	934	1,250	314	153
Third	1,107	928	1,230	348	145
Fourth	1,372	1,051	1,528	475	152
Insured	3,853	3,280	4,390	1,110	537
Employed	2,592	1,753	2,785	1,031	193
Highest education level					
Less than college	3,116	2,522	3,512	990	396
Baccalaureate degree	700	550	781	232	81
Graduate degree	797	646	899	254	102
Region					
Northeast	834	676	949	273	115
Midwest	968	840	1,087	247	119
South	1,792	1,424	2,011	588	220
West	1,046	796	1,178	382	132
Metropolitan area	3,873	3,063	4,357	1,294	484
Total	4,639	3,735	5,224	1,489	587

Notes: Population analyzed is 202 million non-diabetic, non-pregnant adults age 18 or older in the US in 2010. Body weight status is defined by body mass index (BMI): overweight (25 ≤ BMI < 30) and obese (30 ≤ BMI). ADA = American Diabetes Association. USPSTF = US Preventive Services Task Force.

**Table 4:** Characteristics and number of adults with prediabetes detected under ADA and USPSTF guidelines (thousands of people)

**Patient characteristics**	**ADA alone**	**USPSTF alone**	**Either ADA or USPSTF**	**ADA but not USPSTF**	**USPSTF but not ADA**
Age category					
<45	9,973	3,706	10,324	6,617	351
45-54	7,826	4,995	8,557	3,562	731
55-64	8,057	6,378	9,204	2,826	1,147
65+	8,071	9,328	11,233	1,905	3,162
Sex					
Men	19,378	12,977	21,720	8,743	2,342
Women	14,549	11,430	17,598	6,168	3,049
Race and ethnicity					
Non-Hispanic white	19,228	17,250	23,126	5,876	3,898
Non-Hispanic black	6,206	3,477	6,799	3,322	593
Non-Hispanic other	1,122	1,297	1,613	316	491
Hispanic	7,371	2,383	7,779	5,396	408
Body weight status					
Overweight	17,971	9,536	17,971	8,435	0
Obese	15,957	9,481	15,957	6,476	0
Diagnosed hypertension	19,016	24,407	24,407	0	5,391
Household income quartile					
First	7,078	4,830	8,338	3,369	1,133
Second	7,487	5,572	8,828	3,118	1,308
Third	8,336	6,338	9,568	3,509	1,344
Fourth	10,952	7,668	12,584	4,914	1,606
Insured	26,977	21,133	31,827	10,694	4,850
Employed	22,269	13,307	24,399	11,092	2,129
Highest education level					
Less than college	22,342	15,903	25,883	9,979	3,540
Baccalaureate degree	5,414	3,870	6,210	2,339	796
Graduate degree	5,957	4,500	6,964	2,463	1,007
Region					
Northeast	6,006	4,421	7,051	2,630	1,045
Midwest	6,894	5,541	8,010	2,469	1,116
South	13,225	9,335	15,285	5,950	2,060
West	7,803	5,110	8,972	3,862	1,169
Metropolitan area	15,297	10,904	17,873	6,970	2,576
Total	33,927	24,407	39,318	14,910	5,391

Note: Population analyzed is 202 million non-diabetic, non-pregnant adults age 18 or older in the US in 2010. Body weight status is defined by body mass index (BMI): overweight (25 ≤ BMI < 30) and obese (30 ≤ BMI). ADA = American Diabetes Association. USPSTF = US Preventive Services Task Force.

ADA guidelines would detect nearly 1.5 million diabetes cases that would be missed under USPSTF guidelines (Table 3). USPSTF guidelines would detect 587,000 diabetes cases that would be missed under ADA guidelines in the initial year of fully implementing the latter guidelines, but these include adults over age 45 who would be screened in subsequent years. Under the ADA guidelines, within three years all adults age 45 and older would be screened.

ADA guidelines identify substantially more individuals in minority populations as having diabetes and prediabetes than do USPSTF screening guidelines. ADA guidelines detect 40% more blacks and more than twice as many Hispanics with diabetes relative to USPSTF guidelines (Table 3). Nearly 80% more blacks and more than three times as many Hispanics would be detected with prediabetes using ADA guidelines compared to USPSTF guidelines (Table 4). Among Hispanics, USPSTF guidelines miss 5.4 million people with prediabetes who would be detected using ADA guidelines. In comparison, ADA guidelines would miss only 408,000 with prediabetes that would be detected using USPSTF guidelines.

USPSTF guidelines both screen and detect a significantly older population. Whereas 35% of people with diabetes detected under ADA guidelines are age 65 and older, 46% of people detected under USPSTF guidelines are age 65 or older.

### Health care use patterns and risk for undiagnosed diabetes or prediabetes

The above MEPS analysis illustrates the number of people in the US who could be screened and for whom diabetes or prediabetes could be detected applying USPSTF and ADA guidelines population-wide. In general, though, screening occurs opportunistically when patients visit a health care provider during an office visit, outpatient or emergency visit, or when hospitalized. Therefore, we analyzed the health care use patterns for people at high risk for undiagnosed diabetes or prediabetes—in particular, patterns of visiting a primary care provider in 2010—to understand what segment of the population had the opportunity to be screened during that year.

Across the entire US population of adults without diagnosed diabetes and excluding pregnant women, our MEPS analysis suggests that 67% of visits within the health care system were office visits to specialist providers; 22% were visits to primary care providers; and the rest consisted of hospital outpatient visits (7%), emergency department visits (3%) and inpatient hospitalizations (1%).

Strikingly, we found that having a greater number of annual visits within the health care system is positively correlated with a higher probability of undiagnosed diabetes, suggesting that multiple opportunities for diabetes detection are being missed (Table 5). For example, adults having a less than 5% probability for undiagnosed diabetes averaged 4.7 visits in 2010; adults with 5%-10% probability averaged 8.8 visits; and adults with a greater than 10% probability of undiagnosed diabetes averaged 10.7 visits. Adults meeting the ADA screening criteria averaged 6.9 visits, while adults meeting the USPSTF screening criteria averaged 9.1 visits. For comparison, people with diagnosed diabetes averaged 11.7 visits. While averages can be sensitive to outliers, across all the care delivery settings modeled there is a clearly observed relationship between patient probability of undetected diabetes or prediabetes and increasing average annual visits.

**Table 5:** Health care use patterns by predicted undetected diabetes and prediabetes status

		**Average annual visits**					
**Scenario**	**Population in 2010**^**1 **^**(in thousands)**	**Primary care office visits**^**2**^	**Non-primary care office visits**^**3**^	**Outpatient visits**^**4**^	**Emergency visits**^**4**^	**Hospital admissions**^**4**^	**Total**
*Predicted probability of undetected diabetes*							
<0.05	155,511	1.00	3.19	0.29	0.13	0.05	4.67
0.05 to <0.10	37,462	2.01	5.72	0.67	0.21	0.15	8.76
≥0.10	10,652	2.39	6.32	1.51	0.22	0.22	10.67
*Predicted probability of undetected prediabetes*							
<0.10	13,904	0.93	2.62	0.16	0.11	0.03	3.85
0.10 to <0.15	21,019	0.69	2.09	0.19	0.13	0.03	3.12
0.15 to <0.20	19,964	0.67	2.69	0.24	0.11	0.03	3.74
0.20 to <0.25	17,790	0.86	2.79	0.22	0.15	0.04	4.06
0.25 to <0.30	21,049	0.97	3.45	0.40	0.14	0.06	5.01
0.30 to <0.35	17,511	1.26	4.12	0.72	0.15	0.09	6.34
0.35 to <0.40	23,528	1.59	4.72	0.46	0.18	0.10	7.05
≥0.40	68,860	1.75	4.91	0.59	0.18	0.13	7.56
ADA	86,292	1.59	4.39	0.58	0.18	0.11	6.85
USPSTF	59,064	2.14	5.77	0.79	0.23	0.16	9.09
*Diagnosed diabetes*	*20,458*	*2.81*	*7.21*	*1.08*	*0.31*	*0.28*	*11.69*

Note: Analysis of the 2010 Medical Expenditure Panel Survey. ^1^Population analyzed is 202 million non-diabetic, non-pregnant adults age 18 or older in the US in 2010. Representative sample of the non-institutionalized population in the US excluding pregnant women. ^2^Office visit to a general or family practice or general internal medicine practice. ^3^Visits to non-primary care providers (excluding obstetrician-gynecologist visits). ^4^Hospital outpatient or emergency visit, or hospitalization for any reason.

### Opportunities to detect diabetes during primary care office visits

We estimate that the US adult population without diagnosed diabetes made approximately 256 million visits to a primary care provider in 2010. In addition, 58% (50 million people) of adults meeting the ADA diabetes screening criteria had at least one primary care visit in 2010, and among these were an estimated 3.1 million patients with undiagnosed diabetes and 20.4 million with undetected prediabetes (Table 6). Of those adults meeting the USPSTF criteria, 70% (41.5 million people) had at least one primary care visit in 2010, and among these were 2.8 million cases of undiagnosed diabetes and 17.3 million cases of undetected prediabetes.

**Table 6:** Opportunities for screening during primary care office visits

**Applying screening guidelines**	**Meets ADA guidelines**	**Meets USPSTF guidelines**
Number of patients with at least one primary care visit	50,180,000	41,530,000
Percent of patients with at least one primary care visit	58.2%	70.3%
Potential cases of diabetes to detect	3,083,000	2,753,000
Potential cases of prediabetes to detect	20,386,000	17,263,409

Note: Estimates derived from analysis of the 2010 Medical Expenditure Panel Survey using predicted probability of patient having undetected diabetes or prediabetes for 202 million non-diabetic, non-pregnant adults age 18 or older in the US in 2010. Primary care visits identified as visits to a general or family practice or general internal medicine practice. Total primary care visits in 2010 by adults age 18 and older (excluding pregnant patients and patients with diagnosed diabetes) is 256.3 million.

Of the estimated 4.6 million adults with undiagnosed diabetes meeting the ADA screening criteria, 66% could have been identified in 2010 if the criteria had been applied during visits to a primary care provider. Of the 3.7 million adults in the US who have undiagnosed diabetes and who meet the USPSTF screening criteria, 74% could have been identified in 2010 if those criteria had been applied during visits to a primary care provider. For comparison, the CDC estimates that 1.9 million people in the US are newly diagnosed with diabetes each year [5], suggesting that large numbers of asymptomatic adults who meet screening criteria are not being tested.

## Discussion

In this study, we compared two strategies to diagnose currently undiagnosed cases of prediabetes and type 2 diabetes. Our research aimed to answer two sets of questions: (1) How many people in the US in 2010 could have been screened and identified with diabetes and prediabetes under ADA and USPSTF guidelines, and what are the characteristics of populations detected with prediabetes and diabetes? (2) What are the health care use patterns of adults at high risk for prediabetes or diabetes, and how does this affect the ability of primary care providers to implement USPSTF and ADA guidelines? These findings have implications for identifying the most efficient screening strategies.

Comparison of ADA and USPSTF guidelines to address the first set of questions suggests three key implications:

1. ADA guidelines detect more people with diabetes and prediabetes compared to USPSTF guidelines, but the latter require slightly fewer people to be screened for each diabetes case detected. ADA guidelines would screen 19 adults for each case of diabetes detected, whereas USPSTF would screen 16 adults for each case detected. In other words, ADA appears to be more effective, while USPSTF may be more efficient. The relative efficiency of the two alternative strategies will depend on the costs of screening and the net effect of screening on medical care costs. Screening costs and forecasts of medical cost savings from alternative screening strategies are beyond the scope of the current paper. It is important to note, however, that the detection rates of the two screening options considered here are not very different when considering both diabetes and prediabetes. ADA guidelines result in 2.2 people screened per case detected while USPSTF guidelines result in 2.1 people screened per case detected. Because ADA guidelines screen more people, ADA guidelines identified 38.9% more undetected cases of prediabetes and 24.3% more undetected cases of type 2 diabetes than did the USPSTF guidelines.

2. ADA guidelines detect more people with diabetes and prediabetes among racial and ethnic minorities and low-income households that historically have had less access to the health care system.

3. The population detected under ADA guidelines is younger than the population detected under USPSTF guidelines. The health, economic, and quality of life implications of early detection and intervention among younger populations could be quite different from detection among older populations. The lifetime direct medical costs of diabetes for men and women diagnosed between the ages of 25–44 are more than twice the lifetime cost of people diagnosed after age 65—reflecting the fact that people who develop diabetes at earlier ages have more time to develop complications and incur diabetes-related costs [30]. Such findings suggest the potential for substantial lifetime economic benefits to detection and treatment of diabetes at younger ages.

On the question of health care use patterns, our overall findings have two key implications. Our study identified two unique target populations for diagnostic testing: (1) a population in poor health with many contacts with the health care system but no apparent diagnostic testing for prediabetes or type 2 diabetes; and (2) a population who, regardless of health status, had no apparent contact with the health care system and therefore no opportunity for diagnostic testing.

1. The first population represents a missed opportunity, especially given the number of primary care visits reported by these patients. We will leave to others whether this problem is best addressed through increased patient or provider education, changes in preventive screenings, or other combination of strategies. What is clear is that the missed opportunity is substantial.

2. The second population, those without contact with the health care system, will require a more innovative approach. Strategies would need to be tested and further research conducted to better identify these people. For example, is their lack of contact due to a lack of health insurance coverage, low income, or cultural reasons? A more refined identification of these people will allow more effective strategies to be developed. One thing is clear: the traditional office-based approach will not work for people who seldom visit a doctor’s office.

### Study limitations and areas for future research

This study takes advantage of large, nationally representative data sources to simulate the likely screening and detection implications of ADA and USPSTF screening criteria. The regression models that quantify the relationship between patient characteristics and probability of undiagnosed diabetes and prediabetes show strong goodness of fit, and validation activities suggest the models are robust.

One limitation of this study is the omission of some diabetes risk factors (such as a history of gestational diabetes or family history of diabetes) due to a lack of data in the MEPS. Diagnosed prediabetes is excluded as an explanatory variable from our predictive model because diagnosed prediabetes status is unavailable in the MEPS.

Another limitation is that NHANES does not have follow-up testing, so we were unable to model the risk of false positives or false negatives from screening [31]. The estimated prediction equations, though, are designed to identify population subsets that are at high risk for undetected prediabetes or undiagnosed diabetes (rather than identify individual people who should be screened).

This study uses multiple diagnostic tests in NHANES (HbA1c, FPG, and OGTT) to identify people with undetected diabetes and prediabetes for use in the logistic regression analysis. Almost 100% of the sample received an HbA1c test, half (48%) received an FPG test, and 31% received an OGTT test. The CDC (2011 Diabetes Fact Sheet) uses either FPG or HbA1c in the prediabetes or diabetes range to estimate national prevalence of prediabetes and undiagnosed diabetes—stating that HbA1c and FPG are used because these tests are most often used in clinical practice [5]. CDC notes that use of all three tests, a subset of tests, or individual tests produces different estimates of total prevalence of diabetes and prediabetes.

We conducted sensitivity analyses on the predictive model goodness of fit and use of different diagnostic tests to define prediabetes and undetected diabetes for the regression analysis. We find that use of all three diagnostic tests to define diabetes or prediabetes status produced slightly higher regression intercept estimates than using HbA1c and FPG, but produced similar estimates of odds ratios and prediction outcomes. (We scaled each individual’s predicted probabilities of undiagnosed diabetes and prediabetes by 0.975 so that national totals matched CDC’s national estimates for 2010 of 79 million with prediabetes and 7 million with undiagnosed diabetes.) Using only HbA1c to define diabetes or prediabetes status produced lower regression intercept estimates and a stronger age and racial/ethnic minority effect on probability of prediabetes or undetected diabetes. In terms of overall study findings, using prediction equations based only on HbA1c identified an older population with prediabetes and undiagnosed diabetes than reported in this paper. CDC notes that “Research is ongoing to ascertain the best use of laboratory blood tests to detect people who may have prediabetes and to improve the understanding of who has prediabetes” [5].

While this analysis focused on the potential to detect diabetes and prediabetes cases in 2010, the full implications of implementing ADA guidelines would take more than one year to manifest, as ADA guidelines call for asymptomatic adults age 45 without risk factors to be screened every three years.

Future research might explore the health, economic, and quality of life implications of detecting diabetes among different subsets of the population (such as younger versus older adults) to better understand the implications of alternative screening guidelines that differ in their ability to detect diabetes and prediabetes among select populations.

Because patients with risk factors for diabetes (such as obesity and hypertension) tend to have greater medical needs, a disproportionate number of patients seeking care in some settings, such as hospital emergency departments, are likely to be at high risk for undiagnosed diabetes or prediabetes. Work by Silverman et al., for example, suggests the prevalence of undiagnosed diabetes and undetected prediabetes among patients admitted to emergency departments for acute illness is 10.5% and 31.9%, respectively [32]. Still, a large study of 2,260 individuals diagnosed with type 2 diabetes found that 88.3% were diagnosed by a family doctor/general practitioner, 4.4% by an endocrinologist, 0.5% by a cardiologist, 0.7% by a neurologist, and 6% by another specialist [33]. This finding highlights the importance of primary care providers in diagnosis of diabetes.

Future research might explore in more depth the health care use patterns of people at high risk for undiagnosed diabetes and why, despite the high volume of care being provided, there are still many people whose diabetes and prediabetes remains undiagnosed.

While this study shows that USPSTF guidelines are slightly more efficient in identifying people with diabetes (in terms of number of people screened to detect each case of diabetes), ADA guidelines are more effective in terms of identifying more people. Ongoing research to investigate the cost-effectiveness of ADA versus USPSTF screening guidelines, in terms of the cost to screen and the cost of intervention among the prediabetic population to prevent or delay diabetes onset and sequelae, would be an essential component for policy decisions in this area.

## Conclusions

Early detection is the first step to provide counseling and well-organized, evidence-based intervention to prevent or delay the onset of diabetes among those with prediabetes, and to prevent or delay the onset of complications among people with diabetes. Relative to USPSTF guidelines, ADA guidelines identify more people with prediabetes and undiagnosed diabetes (especially more minority cases) and a younger population that allows for the potential for more effective improvements in quality of life and potentially improved outcomes. The health care use patterns of people at high risk for undiagnosed diabetes and prediabetes combined with the high prevalence of undiagnosed cases suggest that many opportunities for diagnosis are being missed. Many high-risk adults do not receive regular care from a primary care provider, which can hamper detection efforts. As health care system technology, health care use patterns, and medical practice continue to evolve, more effective and efficient methods and criteria for diabetes screening in asymptomatic adults should be sought.

## Abbreviations

ADA: American Diabetes Association; FPG: Fasting plasma glucose test; HbA1c: Hemoglobin A1c test; MEPS: Medical Expenditure Panel Survey; NHANES: National Health and Nutrition Examination Survey; OGTT: Oral glucose tolerance test; USPSTF: United States Preventive Services Task Force.

## Competing interests

KG is employed by Novo Nordisk Inc. The authors have no other competing interests.

## Authors’ contributions

TD is the guarantor of this work and, as such, had full access to all the data in the study and takes responsibility for the integrity of the data and the accuracy of the data analysis. TD wrote the manuscript. KMVN, KG, WQ, MO and TB contributed to the discussion and reviewed/edited the manuscript. PG, TB, and MS researched data and contributed to the discussion. TD, KMVN, MS, and PG conceptualized or conducted the statistical analyses. All authors read and approved the final manuscript.
